# A combination hepatoma-targeted therapy based on nanotechnology: pHRE-Egr1-HSV-TK/^131^I-antiAFPMcAb-GCV/MFH

**DOI:** 10.1038/srep33524

**Published:** 2016-09-19

**Authors:** Mei Lin, Junxing Huang, Xingmao Jiang, Jia Zhang, Hong Yu, Jun Ye, Dongsheng Zhang

**Affiliations:** 1 Taizhou People’s Hospital Affiliated to Nantong University, Taizhou, 225300, China; 2 Medical School of Southeast University, Nanjing, 210009, China; 3 Key Laboratory of Advanced Catalytic Material and Technology, Changzhou University, Changzhou, 213000, China; 4 Southeast University, Jiangsu Key Laboratory For Biomaterials and Devices, Nanjing, 210009, China

## Abstract

Combination targeted therapy is a promising cancer therapeutic strategy. Here, using PEI-Mn_0.5_Zn_0.5_Fe_2_O_4_ nanoparticles (PEI-MZF-NPs) as magnetic media for MFH (magnetic fluid hyperthermia) and gene transfer vector for gene-therapy, a combined therapy, pHRE-Egr1-HSV-TK/^131^I-antiAFPMcAb-GCV/MFH, for hepatoma is developed. AntiAFPMcAb (Monoclonal antibody AFP) is exploited for targeting. The plasmids pHRE-Egr1-HSV-TK are achieved by incorporation of pEgr1-HSV-TK and pHRE-Egr1-EGFP. Restriction enzyme digestion and PCR confirm the recombinant plasmids pHRE-Egr1-HSV-TK are successfully constructed. After exposure to the magnetic field, PEI-MZF-NPs/pHRE-Egr1-EGFP fluid is warmed rapidly and then the temperature is maintained at 43 °C or so, which is quite appropriate for cancer treatment. The gene expression reaches the peak when treated with 200 μCi ^131^I for 24 hours, indicating that the dose of 200 μCi might be the optimal dose for irradiation and 24 h irradiation later is the best time to initiate MFH. The *in vitro* and *in vivo* experiments demonstrate that pHRE-Egr1-HSV-TK/^131^I-antiAFPMcAb-GCV/MFH can greatly suppress hepatic tumor cell proliferation and induce cell apoptosis and necrosis and effectively inhibit the tumor growth, much better than any monotherapy does alone. Furthermore, the combination therapy has few or no adverse effects. It might be applicable as a strategy to treat hepatic cancer.

Comprehensive treatment, a joint application of multidiscipline and (or) multi-method by a specific way in view of their respective characteristics, is a promising cancer therapeutic strategy. It is not a simple overlap together of some therapies, but applied reasonably to make their strengths complementary, leading to a synergistic curative effect. Radiotherapy, chemotherapy, thermotherapy and biotherapy all play important roles in cancer treatment, but each has its own merits and demerits and none of them can thoroughly kill cancer cells. It is therefore significant to combine them organically to treat cancer.

Radiotherapy is currently well accepted as one of the most effective remedies for cancer. Radionuclide particularly exhibits a great anticancer potential since it can be selectively delivered to lesion by labeling with special materials such as monoclonal antibody, bioactive peptide, etc., leading to a maximum biological effect and damage as little as possible to normal tissue^1^,^2^,^3^,^4^,^5^. Moreover, nuclide internal irradiation belongs to prolonged low dose rate exposure, but the total cumulative dose can get larger. This facilitates therapeutic genes sustaining expression in radiation-gene therapy while nuclide plays radiotherapy role. But external exposure and internal irradiation are both mainly effective to G2/M phase cells and neither of them has effect on S and G0 phase cells and hypoxic cells, which commonly results in radioresistance. Encouragingly, combination of two methods or more is often superior to any single scheme^6^,^7^,^8^,^9^,^10^. It is reported that both short-term and long-term (survival rate of six months, one year and two years) curative effects (CR + PR) of radiotherapy combined with chemotherapeutics against cervical cancer, lung cancer, and esophageal squamous cell carcinoma and nasopharyngeal carcinoma are significantly better than those of any regime alone^11^,^12^,^13^,^14^,^15^.

Gene therapy is one of important biological treatments, and suicide genes especially show a good application prospect in the field of cancer treatment^16^. In suicide gene therapies, herpes simplex virus type thymidine kinase (HSV-TK) gene is most commonly used. It can express thymidine kinase to transform non-toxic prodrug ganciclovir (GCV) into toxic GCV-TP to kill tumor cells. A large number of studies have proved the anticancer effects of HSV-TK/GCV system, and better curative effects can be achieved when it is combined with radiotherapy^17^,^18^. However, two crucial problems must be solved to accomplish comprehensive gene therapy against cancer for a desired curative effect. One is how to ensure safe gene delivery into cells with high transfection efficacy. The other is how to make gene express efficiently and controllably.

Honestly, radiation-gene therapy shows a promising anticancer prospect. The promoter of Egr1, a transcription factor regulating early cell growth, can induce the expression of its downstream genes after ionizing radiation, thereby attaining a spatio-temporal regulation on the target gene expression. Notable achievements in radiation-gene therapy have been obtained by exploiting the radiosensitivity of Egr1 promoter^19^,^20^,^21^. In addition, hypoxia response element (HRE), an enhancer sensitive to hypoxia, can promote its downstream genes to express in hypoxia environment^22^,^23^,^24^. Shibata *et al*.^25^ has confirmed that five copies of HRE connected with a promoter can increase its downstream gene expression by 500 times in hypoxia condition. In our previous studies, we obtained anoxic radiation double sensitive promoter HRE/Egr1 by coupling Egr1 promoter with HRE and constructed pHRE-Egr1-EGFP plasmids. The resulting HRE/Egr1 could effectively induce and improve EGFP expression in cancer anoxic microenvironment when radiated and the expression level of target gene induced by anoxia and radiation was significantly higher than by radiation alone^17^. However, this method has yet to solve the common problem how to safely and effectively deliver genes into cells in gene therapy.

Two of the major gene delivery vectors, viral vector systems and non-viral vector systems, are currently being used, but both have their own pros and cons. Although viral vectors have been proved efficacious, their small gene capacity, poor target specificity, self-immunogenicity, and especially serious biosafety concerns limit their further application. Despite avoiding the major security risks, non-viral vectors such as lipofection, polyamidoamine^26^, poly(ethyleneimine)^27^, and poly(amino acid)^28^ are greatly inferior to viral vectors in transfection efficiency, and meaningful expression of target gene is hardly available in this system. Therefore, the gene delivery remains a challenge because of the lack of suitable vectors.

It is encouragingly that nanocarriers are emerging as a kind of powerful delivery tool in gene therapy. Such vectors retain the advantages of viral and non-viral carriers and remove their disadvantages and therefore become a new promising carrier system. Compared to traditional carriers, nanocarriers have much superiority, including slowly released the genes, maintained effective concentration of the product, improved transfection efficiency and the product bioavailability. Also nanocarriers have no immunogenicity, no genetic toxicity, no cytotoxicity, and no cell transformation or death^29^,^30^,^31^,^32^,^33^,^34^,^35^,^36^,^37^,^38^. Particularly, except the general properties of nanoparticles, super-paramagnetic magnetic nanoparticle gene transfer vectors can produce highly efficient transfection and do directional movement in an external magnetic field, in turn which help to implement targeting gene therapy. Moreover, since magnetic nanoparticles can generate heat by magnetic induction in an external magnetic field, they also can be used for cancer thermotherapy^39^,^40^,^41^.

Representing an alternative treatment for cancer, thermotherapy not only can play an anticancer role itself, but also can improve the sensitivity of chemotherapy and radiotherapy^42^,^43^,^44^,^45^. It is investigated that when heated for 30 min at 43 °C, the cytotoxicity of paclitaxel (a chemotherapy drug) can increase by 10–100 times, and the killing ability of some chemotherapeutics with little cytotoxicity at normal temperature can double^46^,^47^. Wang etc.^48^ used radiotherapy combined with thermotherapy to treat advanced lung cancer. The effective rate reached 70% (21/30), while the single-radiotherapy was only 44% (14/32). However, there is an intractable technical difficulty in heating tumor tissue evenly at a desired temperature, with no damage to normal tissue during thermal treatment.

Inspiringly, Jordan, *et al*.^49^ developed magnetic fluid hyperthermia (MFH), a new tumor thermotherapy by combining magnetic induction heating with nanotechnology. This therapy has a targeted position function. In other words, in an external magnetic field, the temperature can specifically rise in the tumor tissue containing magnetic nanoparticles, whereas normal tissue without magnetic particles is not subject to thermal damage. It therefore has a great specificity for cancer treatment. We have previously prepared thermo-sensitive Mn-Zn ferrite magnetic nanoparticles (Mn_0.5_Zn_0.5_Fe_2_O_4_, MZF-NPs)^17^,^18^. Both *in vitro* and *in vivo* experiments confirmed their excellent magnetic responsibility, good heating and thermostatic ability, and good biocompatibility^50^,^51^,^52^,^53^,^54^. One of the best performances for MZF-NPs is that they can get a set Curie temperature by adjusting the concentration of their magnetic fluid. To be specific, MZF-NPs are strong magnetic materials below the Curie temperature, which can absorb electromagnetic waves to warm up in an alternating magnetic field. Upon reaching the Curie temperature, they will change into non-magnetic materials and lose the ability to absorb electromagnetic waves, so their temperature starts to decrease. After the temperature falling below the Curie temperature, the materials begin magnetic heating again. So cyclically, the temperature always fluctuates at the set Curie temperature, such as 42–44 °C, which is an effective treatment temperature for tumors, inflicting no damage to normal tissue. It thus successfully resolves the problem of temperature controllability in hyperpyrexia treatment, improving the stability and security of thermotherapy^17^,^55^. Magnetic induction heating by using Mn_0.5_Zn_0.5_Fe_2_O_4_ nanoparticles as magnetic media treated liver cancer and cervical cancer and yielded good outcomes, especially presenting an obvious synergistic effect when combined with As_2_O_3_^56^,^57^. Meanwhile, MZF-NPs modified with PEI have good loading capability, transfect capability and protection ability to DNA and can be used as gene-transfer vectors in gene therapy^17^,^18^,^52^.

In addition to therapeutic effect, good targetability is also crucial for tumor treatment^58^,^59^,^60^. Especially, the progress of radionuclide therapy largely relies on the development of nuclide targeting system. To select an ideal vector with high specificity, adhesion, penetration and carrying capacity to selectively deliver radionuclide into tumor is the key to improve nuclide tumor uptake and T/NT (the ratio of radionuclide in tumor versus in nontumor), and this is also considered as a precondition for nuclide therapy to obtain good curative effects, with an exception of radioiodine to treat thyroid.

As a class of specific tumor antigen in the cell membrane or cytoplasm, α-fetoprotein (AFP) is positive in over 70% of primary hepatic carcinomas, but negative in normal liver or other tissues^61^,^62^,^63^. Consequently, it is a good potential antigen for hepatoma-targeted treatment. Owing to its high specificity and affinity in liver cancer cells, monoclonal antibody AFP (antiAFPMcAb) can be used to carry various “warheads” such as chemotherapy agent, radioactive nuclide or toxin to selectively attack AFP-positive cancer cells^64^,^65^. In our previous study, we prepared radionuclide immune albumin nanospheres (^131^I-antiAFPMcAb-GCV-BSA-NPs, short for ^131^I-antiAFPMcAb-GCV). In these nanospheres, GCV was encapsulated in bovine serum albumin (BSA) nanoparticles. AntiAFPMcAb labeled with radioactive ^131^I was grafted to GCV-BSA nanoparticles to enhance the targetablity of ^131^I and GCV to tumors with over-expressed AFP. The *in vitro* and *in vivo* targeting experiments showed good selectivity of ^131^I and GCV for AFP-positive tumors^66^.

In the present study, we developed a combined therapy, pHRE-Egr1-HSV-TK/^131^I-antiAFPMcAb-GCV/MFH, for hepatoma by using PEI-Mn_0.5_Zn_0.5_Fe_2_O_4_ nanoparticles (PEI-MZF-NPs) as magnetic media for MFH and gene transfer vector for gene-therapy, and investigated the therapeutic effects of pHRE-Egr1-HSV-TK/^131^I-antiAFPMcAb-GCV/MFH on hepatoma *in vitro* and *in vivo*, as well as the underlying safety. As shown in the schematic of the combined therapy (Supplementary Figure 1), since PEI-Mn_0.5_Zn_0.5_Fe_2_O_4_ nanoparticles have good magnetic property and magnetic induction thermogenesis in an alternating magnetic field, they can be used for hyperthermia therapy. While killing hepatoma cells, ^131^I can activate Egr1 promotor to induce HSV-TK gene expression and the expression can be especially enhanced by HRE in hypoxic solid cancer, so the gene therapy can be initiated. The antiAFPMcAb confers the therapy targetability. As a result, a multiple targeting killing of genes, radionuclide and hyperpyrexia against hepatoma can be achieved.

## Results and Discussion

### Characteristics of MZF-NPs

**Figure 1:**
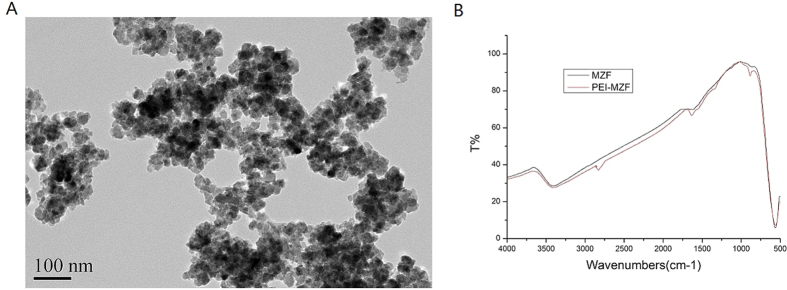
TEM examination of Mn_0.5_Zn_0.5_Fe_2_O_4_ nanoparticles (MZF-NPs) and the infrared spectrum analysis to detect PEI-modified MZF-NPs. (**A**) TEM image of MZF-NPs; (**B**) The infrared spectrum analysis.

The resulting MZF was approximately spherical, about 15–20 nm in diameter and some agglomerate, as revealed by TEM (Fig. 1A). To alleviate the agglomeration of MZF-NPs and make them play roles of gene transfer vector and magnetic induction medium, we modified MZF-NPs with PEI, a surface modification agent, whose monomer (-CH-CH_2_-NH_2_-) has a good ability to bind DNA and adhere to cells^67^. Figure 1B is the infrared spectrum analysis of coated and uncoated MZF-NPs, showing some small characteristic peaks of -NH_2_ and -CH_2_- at 876 cm^−1^, 1316 cm^−1^, 1640 cm^−1^ and 2830 cm^−1^, namely the shear vibration peaks of -NH_2_ of PEI and asymmetric and symmetric stretching vibration characteristic peaks of -CH_2_-, which indicates that MZF-NPs are successfully coated by PEI.

### Identification of pHRE-Egr1-HSV-TK by restriction enzyme digestion

**Figure 2:**
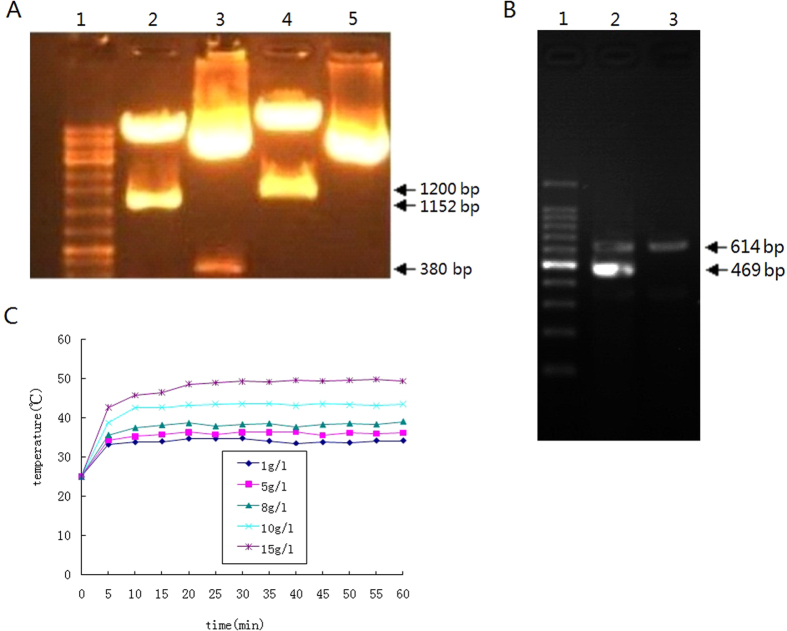
pHRE-HSV-TK was identified by restriction enzyme digestion and HSV-TK expression was examined after transfected into HepG2 cells. And temperature rising of PEI-MZF-NPs combined with pHRE-Egr1-HSV-TK under AMF was tested. (**A**) restriction enzyme digestion of pHRE-HSV-TK (lane 1: marker; lane 2: cut by MluI and NheI; lane 3: cut by BglII; lane 4: cut by EcoRI, XhoI; lane 5: without digestion by any restriction endonuclease). (**B**) HSV-TK expression in HepG2 cells transfected with pHRE-Egr1-HSV-TK (lane 1: Marker; lane 2: HepG2 cells transfected by pHRE-Egr1-HSV-TK group; lane 2: untransfected HepG2 cells group). (**C**) Heating curves of PEI-MZF-NPs/pHRE-Egr1-HSV-TK *in vitro*.

In the constructed pHRE-Egr1-HSV-TK plasmids, there were two BglII restriction sites and the fragment between the two sites contained HRE about 380 bp. The fragment between the two sites on MluI and NheI was Egr1 and the length was about 1152 bp. HSV-TK was inserted between the two restriction sites of EcoRI and XhoI and its length was about 1200 bp. So theoretically, pHRE-Egr1-HSV-TK digested with EcoRI and XhoI can generate an about 1200 bp fragment of HSV-TK. Similarly, MluI and NheI digestion may lead to an about 1152 bp of Egr1 and BglII digestion can result in 380 bp fragment containing HRE. As shown in Fig. 2A, the agarose gel electrophoresis of pHRE-Egr1-HSV-TK digested with EcoRI, XhoI and MluI, NheI and BglII clearly showed a band of about 1152 bp in lane 2, 380 bp in lane 3 and 1200 bp in lane 4. This confirmed the recombinant plasmids of pHRE-Egr1-HSV-TK.

### HSV-TK expression in HepG2 cells mediated by PEI-MZF-NPs

To further verify whether HSV-TK was correctly intercalated into the plasmids, HSV-TK expression was detected by RT-PCR after HepG2 cells were transfected with pHRE-Egr1-HSV-TK by using PEI-MZF-NPs as gene transfer vectors and radiated in hypoxia condition. The findings showed a distinct band of 469 bp for targeted gene TK and internal control GAPDH 614 bp in HepG2 cells/TK, and only one band with 614 bp of GAPDH in the negative control HepG2 cells without transfection (Fig. 2B). These results suggested that HSV-TK was successfully integrated into the plasmids and transfected into HepG2 cells by PEI-MZF-NPs and can express stably at the mRNA level with radiation in hypoxic condition.

### Effects of nuclide dose and radiation time of ^131^I on gene expression

For radiation-gene therapy study, ideally, radiation dose should be large enough to induce gene expression, but not be too high to damage target cells before the gene therapy takes effect. In addition, what time to heat is also important in the comprehensive treatment. In theory, as heat can inhibit the activity of some enzymes, impair DNA damage repair, and increase the permeability of cell membrane to take more drugs into cells, the effect should be better if heating is done firstly or thermotherapy is implemented with radiotherapy or chemotherapy simultaneously. But in the current study, it was radiation-gene therapy combined with thermotherapy that treated hepatocellular carcinoma, if so done, the cells would suffer from thermal damage from the very start, which would weaken the gene expression and consequently lower the effect of gene therapy. To explore the impacts of nuclide dosage and radiation time on gene expression, we checked the report gene EGFP expression in Bel-7402 cells induced by internal irradiation of ^131^I at doses ranging from 50 to 400 μCi within 48 h in hypoxic conditions. The findings showed that EGFP expression had an obvious nuclide dose-effect and time-effect within 24 h and 200 μCi. ^131^I irradiation with 200 μCi for 24 h made gene expression reach the peak. After 24 h or over 200 μCi, the expression started to decline (see Table 1). This indicated that large dose and (or) long irradiation injured the cells and thus weakened the gene expression. Therefore, the dose of 200 μCi might be the optimal dose for irradiation and the best time to initiate thermotherapy was 24 h irradiation later, which can ensure HSV-TK gene express sufficiently and make radionuclide, gene and hyperpyrexia yield a maximum comprehensive effect while each of them playing its own therapeutic role. This provides a theoretical evidence for choosing nuclide dose and when to start heat after irradiation in the later combination therapy.

### Temperature rise of PEI-MZF-NPs/pHRE-Egr1-EGFP *in vitro*

Undoubtedly, heat can improve the sensitivity of radiotherapy and chemotherapy, but the comprehensive curative effect is closely related to heating temperature. The critical temperature leading to death for most tumor cells is 42–43.5 °C, and heating alone at below 42 °C cannot effectively kill tumor cells, even overtime for heating is also of no useful. When temperature is more than 42 °C, thermal damage becomes obviously, and the quantity of killed cells can double when temperature rise by 1 °C^46^,^68^. But when it is more than 45 °C, the thermal sensitivity has no difference between tumor tissue and normal tissue, indicating damage on normal tissue. Therefore, the appropriate temperature for tumor thermotherapy should be over 42 °C, but not more than 45 °C, with an exception of good enough targetability.

In the present study, PEI-MZF-NPs/pHRE-Egr1-EGFP were dispersed in 0.9% NaCl and exposed to a high-frequency alternating electromagnetic field for 60 min. As shown in Fig. 2C, after exposure to the magnetic field, each fluid with different concentration could rapidly warm, and then became stable. As the concentration increased, the top temperature rose. Of them, 10 mg/l fluid rapidly warmed within 5 min, and then gradually reached a steady, the temperature approximately stabilizing at 43 °C or so, which is similar to magnetic induction temperature rise of single PEI-MZF-NPs or MZF-NPs tested in our previous study^16^,^17^, indicating that the combination with DNA does not change MZF-NPs’ magnetic induction temperature rise characteristic. This hyperthermia behavior is quite appropriate for cancer treatment since it can kill tumor cells while not harming normal tissues. Thus, the dose of 10 mg/l was selected for magnetic induction hyperthermia in the later experiments.

### HepG2 cell proliferation tested by MTT

Next, cell proliferation inhibition was explored. The cells of each group with different treatment were incubated over a period of 72 h and the cell viability was determined by MTT. As a classical chemotherapeutic widely used in clinic, adriamycin was chosen as a therapeutic control. As shown in Table 2, the combination greatly inhibited HepG2 cells’ proliferation. The proliferation inhibition rate of cells treated with nuclide-gene-MFH is up to (94.51 ± 0.91)%, significantly higher than (43.91 ± 4.58)% of the nuclide-alone group, (60.33 ± 3.71) of the MFH-alone group, (74.97 ± 1.91) of the nuclide-gene group and (71.68 ± 2.06) of the adriamycin group (*p* < 0.001).

### Flow cytometric analysis of apoptosis and necrosis

**Figure 3:**
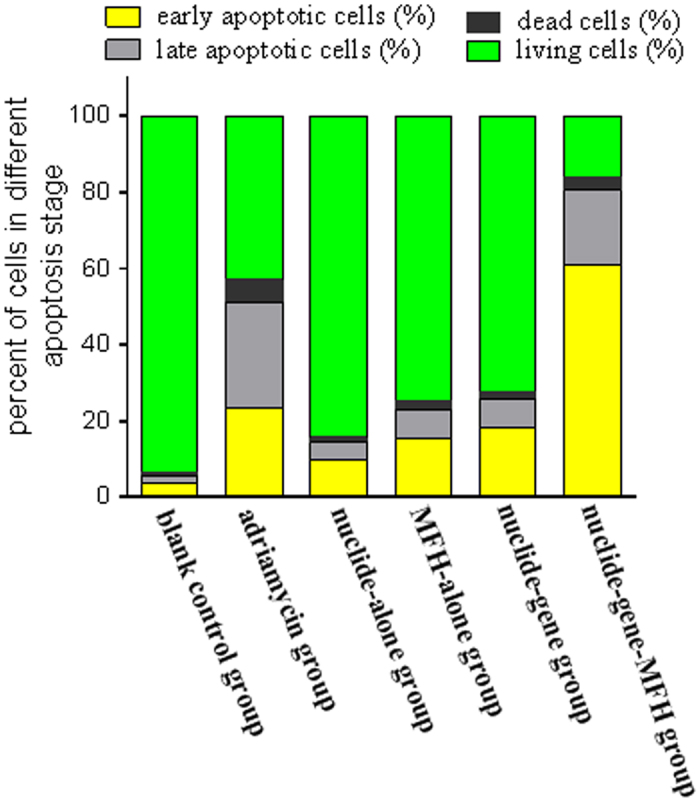
Flow cytometric analysis for apoptosis of HepG2 cells with different treatment (%).

The HepG2 cells of each group after treatment were stained with annexin-V-FLUOS/PI and then analyzed by flow cytometry to detect apoptosis and necrosis. The results showed the best apoptotic and necrotic efficacy in nuclide-gene-MFH group. The total apoptotis and necrosis rate of the nuclide-gene-MFH group reached 84.04%. In contrast, it was only 27.73% in the nuclide-gene group, 15.72% in the nuclide-alone group, 25.22% in the MFH-alone group, 57.03% in the adriamycin group, and only 6.55% in the blank control group (Fig. 3).

### *In vivo* therapeutic effects of pHRE-Egr1-HSV-TK/^131^I-antiAFPMcAb-GCV combined with MFH on xenograft hepatoma in nude mice

To investigate heating effect, antitumor efficacy and safety *in vivo*, hepatoma models were established in subcutaneous tissues of nude mice.

### Heating *in vivo*

Following intratumor injection of PEI-MZF-NPs or PEI-MZF-NPs/pHRE-Egr1-HSV-TK/^131^I-antiAFPMcAb-GCV and exposure to high frequency alternating magnetic field (AMF) for 30 min, the entire tumors almost got heated in MFH-alone group and nuclide-gene-MFH group, and then the tumor temperature maintained at 42–45 °C. All nontumor heating levels were safely below 41 °C during treatment (Supplemental Figure 2).

### The tumor inhibitory effects

After six weeks treatment, all the tumors were removed from the nude mice and measured in volume and weight. Compared with saline control group, the tumors of all therapeutic groups became smaller, but those of nuclide-gene-MFH group showed the smallest. The mass and volume inhibition ratios in nuclide-gene-MFH group were (96.38 ± 1.56)% and (94.17 ± 3.12)% respectively, significantly higher than (35.73 ± 11.60)% and (37.50 ± 11.38)% of nuclide-alone group, (78.11 ± 6.82)% and (76.67 ± 7.41)% of MFH-alone group, (68.07 ± 6.14)% and (75.58 ± 4.43)% of the nuclide-gene group, and (76.66 ± 5.36)% and (77.23 ± 3.84)% of adriamycin group (*p* < 0.001) (Table 3). Seen from *in vivo* tumor growth curves (Supplemental Figure 3), nuclide-gene-MFH group also showed the best therapeutic efficiency. The growth of tumors treated with nuclide-gene-MFH was greatly inhibited, obviously better than that treated with saline and the other therapies.

### Histopathological findings, cell ultrastructural examination and immunohistochemistry (IHC) assays

To further assess the mechanism of therapeutic effects, histology of the excised tumor were analyzed via H&E staining and cell ultrastructure was detected by TEM.

**Figure 4:**
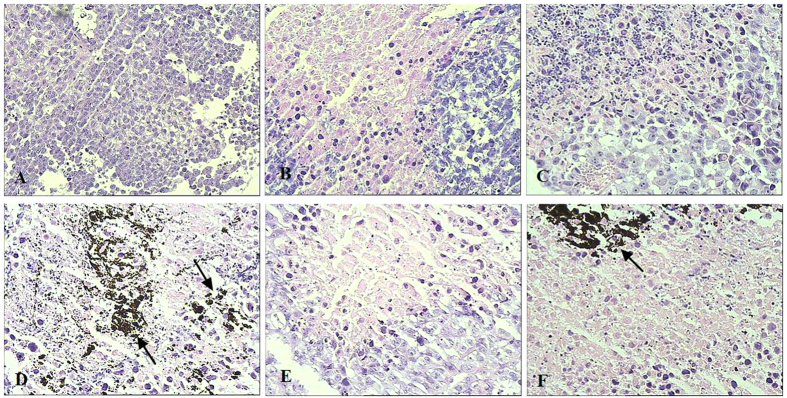
Xenograft hepatoma histopathological findings (stained with HE, ×400). (**A**) saline control group; (**B**) adriamycin group; (**C**) nuclide-alone group; (**D**) MFH-alone group; (**E**) nuclide-gene group; (**F**) nuclide-gene-MFH group.

The H&E staining assays showed some magnetic nanoparticles (marked by the arrows) cumulated in the tumor tissue of MFH-alone group and nuclide-gene-MFH group. Widespread tumour necrosis with nucleus collapse and many inflammatory cells emerged in nuclide-gene-MFH group, much more than that in the other therapeutic groups, while tumor cells in saline control group were dense, with plenty cytoplasm and deep-dyed big nucleus (Fig. 4). These findings were consistent with the results of *in vitro* antitumor assays and *in vivo* tumor growth inhibitory effects.

**Figure 5:**
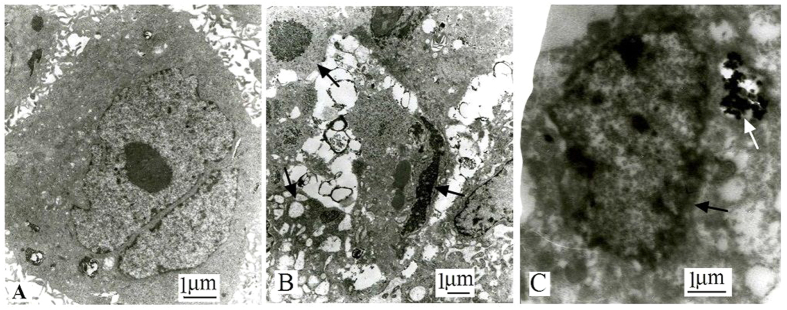
Xenograft hepatoma cells’ ultrastructure observed with TEM. (**A**) saline control group (×10000); (**B,C**) nuclide-gene-MFH group (**B** ×15000, **C** ×15000).

As an active cellular suicide, apoptosis is often characterized as chromatin margination, nuclear fragmentation, cytoplasmic blebbing and internucleosomal fragmentation of DNA. Cell ultrastructure examination by TEM verified that nuclide-gene-MFH effectively induced xenograft hepatoma cells apoptosis. As shown in Fig. 5, hepatoma cells in nuclide-gene-MFH group (Fig. 5B,C) exhibited typical morphologically features of apoptotic cells such as karyopyknosis, chromatin condensation and margination or cleavage, apoptotic body, and cytoplasmic vacuolation (marked by the black arrows), and some magnetic materials (marked by the white arrow) deposited in the cytoplasm. By contrast, hepatoma cells in saline control group (Fig. 5A) were regular shape, with intact nucleus, fine chromatin and large nucleolus.

**Figure 6:**
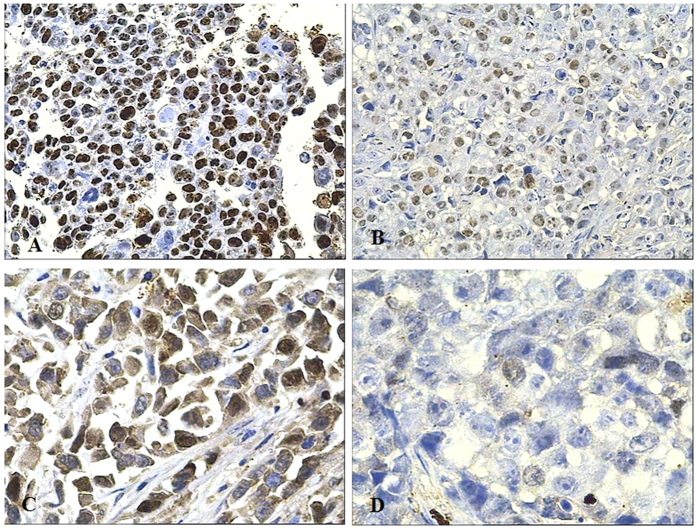
Ki67 and survivin protein expression in xenograft hepatoma tissue tested by IHC. (**A**) Ki67 of saline control group (200×); (**B**) Ki67 of nuclide-alone group (200×); (**C**) survivin of saline control group (400×); (**D**) Survivin of nuclide-alone group (400×).

To further confirm the proliferation inhibition and apoptotic induction of the combination therapy, immunohistochemistry (IHC) assay were applied to detect Ki67 and survivin protein expression. As the strongest apoptosis inhibition factor ever discovered, survivin is closely related to the development and prognosis of cancer. It is barely expressed in normal tissues, but over-expressed in many tumors such as hepatoma, gastric cancer. The over-expressed survivin protein can inhibit apoptotic protease, help tumor cells escape from the checkpoint monitoring of cell cycle G2/M transition and resist cell apoptosis from DNA damage or mutation, resulting in abnormal cell division and proliferation^69^. Ki-67, a nuclear proliferation marker, is one of the most reliable indictors to assess the proliferative activity of tumor cells. It can be applied to evaluate the efficacy of cancer treatment. The more Ki-67 expresses, the brisker the cells grow^70^,^71^. Seen from the IHC assays (Fig. 6 and Table 4), Ki67 protein and survivin protein in the tumor tissue both greatly decreased after nuclide-gene-MFH treatment. In sailing group, Ki67 and survinin positive percent got (79.02 ± 3.58)% and (90.36 ± 3.53)%, respectively, and their positive index both reached 12 which means strong positive. Whereas Ki67 and survinin positive percent in nuclide-gene-MFH group declined to only (10.18 ± 1.64)%, (6.72 ± 1.54)%, respectively, and their positive index were both merely 1 which means extremely faint positive. This finding indicates that this combination may inhibit the tumor cells proliferation via inhibiting Ki67 protein expression and induce tumor cells apoptosis via inhibiting survivin protein expression.

The above datas indicate that pHRE-Egr1-HSV-TK/^131^I-antiAFPMcAb-GCV/MFH may make a good complemental synergetic anticancer effect, reaching 1 + 1 + 1 > 3. The mechanisms of action likely include the followings:^17^ (1) Heat damages the repair of DNA fractured by radionuclide; (2) High temperature destroys the biological integrity of cell membrane and increases its permeability, which is helpful to the chemical drug and gene penetration and absorption; (3) G2 and M phase cells are very sensitive to radiation, whereas S phase cells are resistant to radiation but very sensitive to heat. Thus, nuclide radiotherapy and hyperpyrexia therapy can complement each other; (4) Heat rouses some dormant cells (G0 phase) into proliferation stage through improving the cell metabolism level. These dormant cells are usually resistant to radiation, but can become sensitive to radiation once getting into proliferation stage. As well, the sensitivity of original proliferative cells is further enhanced; (5) Heating can improve the radiation sensitivity of hypoxic tumor cells by improving the blood and oxygen supply to tumor region. Taken together, radionuclide, suicide gene and MFH play distinct roles in antitumor therapy. The three combination may overcome their own flaws and make their strengths complementary, leading to a synergistic effect. In addition, targeted treatment guided by antiAFPMcAb may also contribute to the good therapeutic effect.

### *In vivo* safety evaluation

It is well known that chemotherapy and radiotherapy frequently result in side effects such as liver and kidney function damage, blood cells decrease caused by inhibiting bone marrow hematopoietic, and so on. For suicide gene therapy, non-toxic precursor drugs only can be converted to active substances in the place where suicide genes express, therefore, in theory, as long as the targeting of gene delivery is good enough, the treatment will not cause systemic side effects. In comparison, thermotherapy is considered a relatively safe and reliable therapeutic strategy which patients are nearly able to tolerate. Moreover, heat treatment can improve patient immunity and reduce adverse effects caused by chemotherapy and radiotherapy.

**Figure 7:**
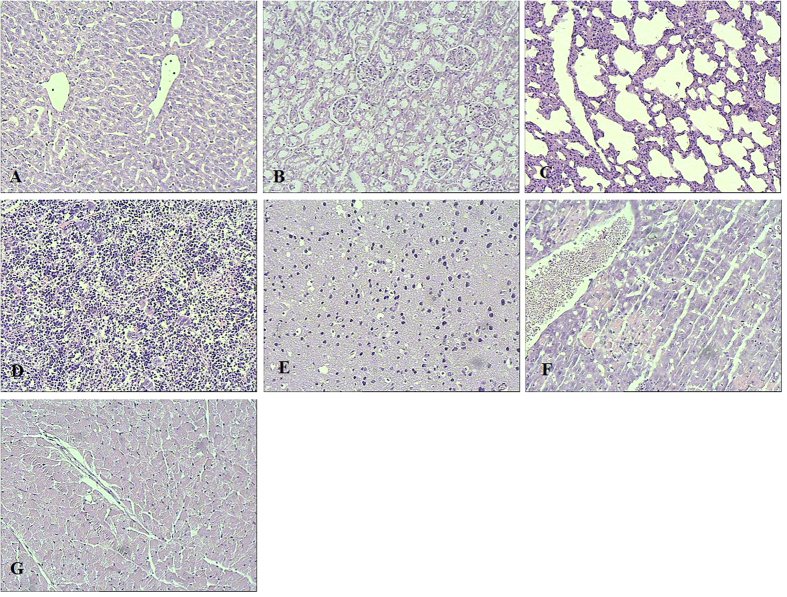
Histopathological findings of some organs from nude mice bearing xenograft hepatoma after different treatment (stained with HE, ×400). (**A**) liver. (**B**) kidney. (**C**) lung. (**D**) spleen. (**E**) brain. (**F**) heart. (**G**) pancreas.

In the present study, the safety *in vivo* of the combination targeted therapy against hepatoma was preliminarily evaluated. We observed the survival of nude mice and tested AST, ALT, BUN and Cr levels in plasma and counted peripheral blood cells of nude mice treated by pHRE-Egr1-HSV-TK/^131^I-antiAFPMcAb-GCV/MFH, compared with those of normal nude mice without tumor, nude mice with tumor but without treatment and nude mice treated with the other therapies. In addition, we examined histological changes of heart, liver, spleen, lung, kidney, brain, and pancrease by H&E staining. We found no mouse death during treatment. AST, ALT, BUN and Cr levels (Table 5) and the quantity of WBC, RBC, PLT (Table 6) did not change appreciably in any group, with no significant differences in the values among the seven groups (*p* < 0.05), suggesting no adverse effects on liver function, kidney function and no inhibition on marrow hematopoiesis of mice. The histological findings also exhibited no obvious pathological abnormalities in heart, liver, spleen, lung, kidney, brain, and pancrease tissues based on H&E staining (Fig. 7). These results indicated that the targeted combined therapy is safe and reliable. Of course, more safety indicators need to be further assessed. All this serves as a scientific theory and a sound basis upon which further tumor treatments and studies in clinic will be conducted in the future.

## Conclusion

In this study, we successfully combined radionuclide, suicide gene and MFH organically to treat hepatoma targetly by using PEI-MZF-NPs as a linker. The *in vitro* and *in vivo* experimental results demonstrate that this combined hepatoma-targeted therapy has a good therapeutic efficacy, far better than any monotherapy and adriamycin chemotherapy. Furthermore, this combined therapy has few or no adverse effects. Thus, it offers a new effective and feasible strategy for hepatoma treatment.

## Material and Methods

### Main materials

DMEM, 0.25% trypsase/0.038% EDTA and fetal bovine serum from Gibco; PEI (polyethylenimine) purchased from Sigma; Adriamycin purchased from Pfizer; agarose purchased from MRI; ^131^I from Nanjing Senke company; Thiazolyl blue (MTT) from AMRESCO; pCDNA3.1-5HRE-Egr1-EGFP (pHRE-Egr1-EGFP)^18^ and pCDNA3.1-Egr1-HSV-TK (pEgr1-HSV-TK)^16^ constructed previously;^16^,^17^ Annexin V-FITC/PI kit purchased from Invitrogen Corporation; Ki67 and survivin immunohistochemistry kits purchased from Cell Signaling Technology Corporation; antiAFPMcAb from Shanghai Yemin biotechnology company; ^131^I-antiAFPMcAb-GCV-BSA-NPs self-prepared;^66^ HepG2 and Bel-7402 cell lines were provided by the Institute of Biochemistry and Cell Biology, Shanghai Institute of Biological Sciences, Chinese Academy of Sciences.

### Preparation, surface modification and characterization of MZF-NPs

MZF-NPs were synthesized by chemical coprecipitation technique according to references^56^,^57^. Their morphology was observed by transmission electron microscopy (TEM) (JEM-200CX, Japan). PEI was used to modify the obtained MZF-NPs^17^,^18^ and fourier transform infrared spectrometry (FTIR) was utilized to analyze whether MZF-NPs were coated by PEI.

### Construction and identification of eukaryotic expression plasmids, pHRE-Egr1-HSV-TK

We have constructed pHRE-Egr1-EGFP^18^ and pEgr1-HSV-TK^17^ in our previous study. To replace EGFP of pHRE-Egr1-EGFP with HSV-TK, pEgr1-HSV-TK and pHRE-Egr1-EGFP were digested by EcoRI and XhoI respectively and then were connected in this study. The connected products were transfected into DH5α. After amplified, separated and purified, the pHRE-Egr1-HSV-TK plasmids was cut by BglII, MluI and NheI, EcoRI and XhoI, respectively, and then were examined by agarose gel electrophoresis. Uncut pHRE-Egr1-HSV-TK served as controls.

### To test the expression of HSV-TK by RT-PCR

To determine the expression of HSV-TK, pHRE-Egr1-HSV-TK was transfected via PEI-MZF-NPs. In detail, PEI-MZF-NPs and pHRE-Egr1-HSV-TK were diluted in their separate serum-free medium (mass ratio of PEI-MZF-NPs/DNA was 40:1), then mixed together and incubated for 30 minutes at room temperature. Subsequently, pHRE-Egr1-HSV-TK/PEI-MZF-NPs complex was obtained. (2) HepG2 cells were seeded in 6-well plates at a density of 5 × 10^5^ cells per well and incubated in routine conditions. About 18 hours later, the original culture medium was discarded, and the cells were rinsed twice with PBS and once with serum-free DMEM. (3) Serum-free DMEM with pHRE-Egr1-HSV-TK/PEI-MZF-NPs was added to the wells (DNA: 3 μg/well), and then the plates continued to be in the couveuse. 5 hours later, the serum-free medium was replaced by fresh serum DMEM medium, and then the cells continued to be incubated. The transfected cells were named HepG2/TK.

After incubation for 24 h, the HepG2/TK cells were exposed to X-ray with 4 Gy (6 Mev) under a linear accelerator (SIEMENS, PRIMUS.HI), then continued to be incubated at 37 °C in hypoxic conditions (0.1% O_2_, 5% CO_2_ and N_2_ balance gas). 72 h later, the total RNA of the HepG2/TK cells was extracted. As well, the HepG2 cells without transfection were used as negative control, and GAPDH cDNA was used as internal reference. Primer sequences were synthesized by the Shanghai Ying Jun Company.

TK-f: CCC ACG CTA CTG CGG GTT TAT (153–174);

TK-r: TGT TGG TGC CGG GCA AGG TC (621–602)

The product length = 469 bp

GAPDH-f: 5′–GCCACATCGCTCAGACAC–3′

GAPDH-r: 5′–CATCACGCCACAGTTTCC–3′

The product length = 614 bp

The system reacted according to one-step method of RT-PCR, and the step was carried out by kit specification. 1% agarose gel electrophoresis was used to identify the results.

### To test effects of nuclide dose and radiation time on gene expression

(1) The Bel-7402 cells were seeded in culture bottles (2 × 10^6^ cells per bottle) and incubated in hypoxic conditions. About 18 hours later, the original culture medium was discarded, and the cells were washed twice with PBS and once with serum-free DMEM. (2) Serum-free DMEM with pHRE-Egr1-HSV-TK/PEI-MZF-NPs was added to the bottles (DNA: 3 μg/bottle, PEI-MZF-NPs: pHRE-Egr1-EGFP = 40:1), and then the bottles were continued to be in the couveuse. 5 hours later, the serum-free medium was replaced with fresh serum DMEM medium, and then the cells were continued to be incubated. (3) After 24 h, 50 μCi, 100 μCi, 150 μCi, 200 μCi, 300 μCi and 400 μCi of ^131^I were added to the corresponding bottle, and the bottles were incubated in hypoxic conditions at 37 °C. (4) 4, 8, 16, 24 and 48 h later, the cells were collected respectively. Flow cytometry were used to test the cells’ fluorescence intensity to screen the nuclide dose and radiation time at the maximum gene expression.

### Heating of PEI-MZF-NPs/pHRE-Egr1-EGFP *in vitro*

1, 5, 8, 10 and 15 g/1 magnetofluids (mass = PEI-MZF-NPs.) were prepared by various doses of PEI-MZF-NPs/pHRE-Egr1-EGFP (The mass ratio of PEI-MZF-NPs and pHRE-Egr1-EGFP was 40:1) being dispersed in 5 ml 0.9% NaCl, respectively. After ultrasonic dispersal, magnetofluids with different concentrations were in turn placed in corresponding flat-bottomed cuvettes under a high-frequency AMF (P = 4 kW; f = 230 kHz; I = 30 A; Ho = 1000 A/m) (SP-04C, Shenzhen, China) for 60 min, with a distance of 5 mm from the bottom of the cuvette to the center of the hyperthermia-coil (coil diameter = 6.5 cm, turn number = 4). The temperature was measured at 5 min intervals. Heating curves were drawn, using the temperature as ordinate and time as abscissa.

### The *in vitro* anti-hepatoma effect of pHRE-Egr1-HSV-TK/^131^I-antiAFPMcAb-GCV/MFH

#### MTT assay for cell proliferation

The cytotoxicity of the combination was estimated in HepG2 cells by MTT assay. After transfected as described above and incubated for 48 h, HepG2/TK cells and HepG2 cells were digested with 0.25% trypsin and diluted into single cell (4 × 10^5^ cells/ml) with the fresh complete medium respectively, and then were seeded in six culture bottles (5 ml/bottle), grouped as (1) untreated group (without transfection), served as a blank control; (2) ^131^I group (without transfection, nuclide-alone group); (3) pHRE-Egr1-HSV-TK/^131^I-antiAFPMcAb-GCV bottle, short for radionuclide-gene group; (4) MFH-alone group (without transfection); (5) pHRE-Egr1-HSV-TK/^131^I-antiAFPMcAb-GCV/MFH bottle, short for radionuclide-gene-MFH group; and (6) adriamycin group. After incubation for 24 h, ^131^I-antiAFPMcAb-GCV-BSA-NPs (final concentration: 200 μCi), PEI-MZF-NPs (final concentration: 10 g/l), ^131^I (final concentration: 200 μCi), adriamycin (final concentration: 10 mg/l) and DMEM were added to the corresponding group, respectively. Group (3) was cultured for 48 h at 37 °C under hypoxic conditions (0.1% O_2_, 5% CO_2_ and N_2_ balance gas). After incubation for 24 h at 37 °C under hypoxic conditions, group (5) were heated for 1 h on a high frequency heater coil plate (4 kw, 230 Hz, 30 A) and then further incubated for 23 h at 37 °C under hypoxic conditions. Group (1), (2), (4) and (6) were cultured for 48 h in routine conditions. Subsequently, the cells in each bottle were digested with 0.25% trypsin and diluted into single cell with their corresponding original culture medium, then some of them were subcultured in 96-well plates (200 μl/well), respectively. Group (3) and (5) were further incubated for 24 h under hypoxic conditions. Group (1), (2), (4) and (6) were ulteriorly cultured for 24 h in air 5% CO_2_ at 37 °C. 24 h later, 20 μl (5 g/l) of MTT was added to the cells in each well and continued to be incubated for 4 h. The culture medium was replaced with 150 μl of DMSO and vibrated for 10 min. Then the optical density (OD) values were measured at a wavelength of 493 nm using a microplate reader (Multiskan MK3-353, USA). The cell proliferation inhibition ratio was calculated with the following formula: proliferation inhibition ratio (%) = (1 −  OD of the experimental group/OD of the blank control group) ×100%. Three replicates were done in every group and each experiment was performed three times.

#### Flow cytometry assay

To evaluate the effect of the combination therapy on cell apoptosis and necrosis, the remaining cells of each group above (for MTT assay) were collected respectively for flow cytometry assay. After wash with PBS, supernatant in each group was decanted and each cell pellet was resuspended in 100 μl staining solution which contained 2 μl Annexin-V-FLUOS, 2 μl propidium iodide (PI) and 96 μl binding buffer, respectively. After 15 min incubated in dark at 25 °C, each cell sample was added with 400 μl binding buffer and then analyzed by a flow cytometer (FCM, Vantage SE, BD Company, USA) within 1 h. Each experiment was repeated three times.

#### *In vivo* experiments of nude micebearing hepatocellular carcinoma

Female BALB/c nude mice, aged 6 weeks, weighing 20–22 gram, purchased from the Lakes Animal Experimental Center of the Institute of Biochemistry and Cell Biology, Shanghai Institute of Biological Sciences, China, were used for the experiments. The experiments were approved by the Animal Care Committee of Jiangsu Province and were performed in accordance with the institutional guidelines. All the mice were maintained in the sterile barrier system of Medical School, Southeast University, China. Exponentially growing HepG2 cells (2 × 10^6^ cells) were injected subcutaneously around the right posterior limb rump.

When tumors reached the desired volume (0.3–0.5 cm^3^), mice were randomly divided into six groups of five mice each: (1) saline group (blank control group), (2) adriamycin group (therapeutic control group), (3) MFH-alone group, (4) ^131^I group (nuclide-alone group), (5) pHRE-Egr1-HSV-TK/^131^I-antiAFPMcAb-GCV group (short for radionuclide-gene group); and (6) pHRE-Egr1-HSV-TK/^131^I-antiAFPMcAb-GCV/MFH group (short for radionuclide-gene-MFH group).

Applied a multipoint intratumor injection strategy, group (1) was injected with sterile saline (0.5 ml/mice); Group (2) was injected with adriamycin (5 mg/mouse); Group (3) was injected with PEI-MZF-NPs fluid (10 g/l, 5 mg/mouse); group (4) was injected with I^131^ (7.4 MBq/mouse); Group (5) was injected with pHRE-Egr1-HSV-TK/PEI-MZF-NPs (pHRE-Egr1-HSV-TK/MZF = 40:1; pHRE-Egr1-HSV-TK: 10 μg/mouse) and ^131^I-antiAFPMcAb-GCV-BSA-NPs (7.4MBq/mouse); Group (6) was injected with pHRE-Egr1-HSV-TK/PEI-MZF-NPs (MZF: 5 mg/mice; pHRE-Egr1-HSV-TK:10 μg/mouse) and ^131^I-antiAFPMcAb-GCV-BSA-NPs (7.4 MBq/mouse). To avoid ^131^I being ingested by the thyroids, enough 1% potassium iodide was added to water drunken by mice involved in ^131^I experiment. 24 h later, the tumors of group (3) and (6) were heated on a high frequency heater coil plate (4 kW, 230 KHz, 30 A) for 1 h after anesthesia with phenobarbital sodium. All the treatments were done three times and the interval was five days. Six weeks later, the blood of mice in each group was extracted by cutting tail and all the mice were sacrificed. Tumors and major organs (heart, liver, spleen, lung, kidney, brain, and pancrease) were collected for further studies. The blood of five normal nude mice without tumor was extracted to be used as a normal control.

### Heating rise *in vivo* detection

During the mice of group (3) and (6) were heated on a high frequency heater coil plate, the temperature of tumor and nontumor region was measured using an infrared thermometer (ZyTemp-TN18 model, China) at 5 min intervals, respectively. Using the temperature as ordinate and time as abscissa, heating curves *in vivo* were drawn.

#### Tumor growth inhibition test

During treatment, the volumes of tumors were measured every 4–7 days, which were calculated as *V* = ab^2^/2 (The a is the long diameter and the b is short diameter). The tumor growth curves *in vivo* were drawn, using treat time as abscissa and tumor volume as ordinate. After treatment, the volume of tumors removed from mice were also measured and calculated as the above formula. In addition, the mass of tumors were weighted. Tumor growth inhibition ratio was evaluated by measuring mass and volume inhibition percentage. Mass inhibition ratio = (1 − relative tumor mass) ×100%, where relative tumor mass was the tumor mass of the experimental group divided by the mean tumor mass of the saline control group. Volume inhibition ratio = (1 − relative tumor volume) ×100%, where relative tumor volume was the tumor volume of the experimental group divided by the mean tumor volume of the saline control group.

#### Histology and cell ultrastructure examination

After mass and volume measurement, some removed tumors were fixed in 4% neutral formaldehyde, then sectioned for HE histopathological examination. Some removed tumors were fixed in 4% glutaraldehyde and then made into ultrathin sections for cell ultrastructure detection by transmission electron microscope (TEM) (JEM-200CX, Japan). Some other removed tumors were embedded in paraffin, then cut into sections (4 μm in thick) for Ki67 and survivin IHC analysis.

To assess the safety, hearts, livers, spleens, lungs, kidneys, brains, and pancreases removed from mice of the radionuclide-gene-MFH group were fixed in 4% neutral formaldehyde and then sectioned for HE histological detection.

#### Blood cells and biochemical examination

To evaluate impacts on marrow hematopoiesis function, white cells (WBC), red cells (RBC) and platelets (PLT) in the blood of mice in each group were counted by an automatic blood cell analyzer (PERLONG MEDICAL, XFA6100, China). After blood samples were centrifuged and separated, alanine aminotransferase (ALT), aspartate aminotransferase (AST), urea nitrogen (BUN) and creatinine (Cr) levels in the plasma were detected by a biochemical autoanalyzer (Beckman- LX20, USA) for liver and kidney function assessment.

#### Statistical analysis

Values are shown as mean ± SD. The data were analyzed with the SPSS 16.0 program. A *p* value of <0.05 was considered significant.

## Additional Information

**How to cite this article**: Lin, M. *et al*. A combination hepatoma-targeted therapy based on nanotechnology: pHRE-Egr1-HSV-TK/^131^I-antiAFPMcAb-GCV/MFH. *Sci. Rep.* **6**, 33524; doi: 10.1038/srep33524 (2016).

## Supplementary Material

Supplementary Information

## Figures and Tables

**Table 1:** EGFP expression in Bel-7402 cells induced by ^131^I (average intensity of fluorescence).

Group	4 h	8 h	16 h	24 h	48 h
50 μCi	66.5	79.3	105.3	88.4	55.6
100 μCi	86.9	98.7	138.9	103.7	88.7
150 μCi	101.6	143.8	179.8	134.8	111.9
200 μCi	183.7	255.9	370.8	495.4	278.9
300 μCi	174.8	145.9	128.8	99.4	76.7
400 μCi	136.5	114.2	77.5	55.3	43.5

**Table 2:** Proliferation inhibiting effect of different treatments on HepG2 cells.

Group	Proliferation inhibition (%, mean ± s, n = 5)
blank control group	0
Adriamycin group	71.68 ± 2.06^a,c,d,f^
nuclide-alone group	43.91 ± 4.58^a,b,d,e,f^
MFH-alone group	60.33 ± 3.71^a,b,c,e,f^
nuclide-gene group	74.97 ± 1.91^a,c,d,f^
nuclide-gene-MFH group	94.51 ± 0.91^a,b,c,d,e^

^a^*p* < 0.000 versus blank control group; ^b^*p* < 0.001 versus adriamycin group; ^c^*p < *0.001 versus nuclide-alone group; ^d^*p* < 0.05 versus MFH-alone group; ^e^*p* < 0.05 versus nuclide-gene group; ^f^*p* < 0.001 versus nuclide*-*gene-MFH group.

**Table 3:** The volume and mass inhibition of hepatoma in nude mice after different treatments.

Group	Tumor volume inhibition rate (%, mean ± s, n = 5)	Tumor mass inhibition rate (%, mean ± s, n = 5)
Adriamycin group	76.66 ± 5.36^2,4,5^	77.23 ± 3.84^b,e^
nuclide-alone group	35.73 ± 11.60^1,3,4,5^	37.50 ± 11.38^a,c,d,e^
MFH-alone group	78.11 ± 6.82^2,4,5^	76.67 ± 7.41^b,e^
nuclide-gene group	68.07 ± 6.14^1,2,3,5^	75.58 ± 4.43^b,e^
nuclide-gene-MFH group	96.38 ± 1.56^1,2,3,4^	94.17 ± 3.12^a,b,c,d^

Tumor volume inhibition rate: ^1^*p* < 0.05 versus adriamycin group; ^2^*p* < 0.001 versus nuclide-alone group; ^3^*p* < 0.001 versus MFH-alone group; ^4^*p* < 0.05 versus nuclide-gene grou*p*; ^5^*p* < 0.001 versus nuclide-gene-MFH group. Tumor mass inhibition rate: ^a^ *p* < 0.05 versus adriamycin control group; ^b^*p* < 0.001 versus nuclide-alone group; ^c^*p* < 0.05 versus MFH-alone group; ^d^*p* < 0.05 versus nuclide-gene group; ^e^*p* < 0.001 versus nuclide-gene-MFH group. These measurements were obtained after treatment for 6 weeks.

**Table 4:** Ki67 and surviving protein expression tested by IHC (mean ± s, *n = *5).

Group	Ki67		Survivin	
	Saline group	Nuclide-gene-MFH group	Saline group	Nuclide-gene-MFH group
positive percent	79.02 ± 3.58	10.18 ± 1.64^a^	90.36 ± 3.53	6.72 ± 1.54^b^
positive index	12	1	12	1

Positive percent (%): ^a^*p* < 0.000 versus saline Ki67 group; ^b^*p* < 0.000 versus saline survivin group.

**Table 5:** Blood cells counting (mean ± s).

Group	WBC (×10^9^/L)	RBC (×10^12^/L)	PLT (×10^9^/L)
normal control group	5.1 ± 5.3	3.0 ± 3.5	362.5 ± 54.3
saline control group	5.2 ± 0.9	3.4 ± 0.4	313.5 ± 69.4
adriamycin group	4.5 ± 0.7	3.6 ± 0.5	317.5 ± 58.4
nuclide-alone group	4.9 ± 0.7	3.2 ± 0.4	363.3 ± 79.4
MFH-alone group	4.7 ± 0.6	3.3 ± 0.7	327.7 ± 70.4
nuclide-gene group	4.7 ± 0.8	3.3 ± 0.8	318.1 ± 65.0
nuclide-gene-MFH group	4.8 ± 0.7	3.2 ± 0.3	326.0 ± 60.7

WBC, RBC and PLT have no differences between all the therapeutic groups and normal control group or saline control group (*p* > 0.05).

**Table 6:** Biochemical examination (mean ± s).

Group	ALT (U)	AST (U)	Bun (mmol/L)	Cr (μmol/L)
normal control group	27.8 ± 6.0	104.1 ± 10.2	4.8 ± 1.2	57.0 ± 4.3
saline control group	28.5 ± 3.9	103.2 ± 8.6	5.6 ± 0.7	55.8 ± 6.1
adriamycin group	33.2 ± 1.6	108.3 ± 5.3	5.5 ± 1.2	52.4 ± 5.7
nuclide-alone group	28.1 ± 3.9	105.4 ± 10.3	5.2 ± 1.2	53.6 ± 6.6
MFH-alone group	28.2 ± 4.8	109.8 ± 8.8	5.3 ± 1.1	55.5 ± 7.6
nuclide-gene group	27.8 ± 3.7	106.6 ± 9.3	5.2 ± 1.2	54.4 ± 7.3
nuclide-gene-MFH group	25.1 ± 4.0	107.2 ± 6.5	5.3 ± 0.8	54.6 ± 3.9

ALT, AST, BUN and Cr have no differences between all the therapeutic groups and normal control group or saline control group (*p* > 0.05).
