# Supplementary material for: A combination hepatoma-targeted therapy based on nanotechnology: pHRE-Egr1-HSV-TK/131I-antiAFPMcAb-GCV/MFH
Source: Sci Rep. 2016 Sep 19;6:33524. doi: 10.1038/srep33524 (PMC5027595; doi:10.1038/srep33524)

Supplementary Information

A combination hepatoma-targeted therapy based on nanotechnology: pHRE-Egr1-HSV-TK/^{131}I-antiAFPMcAb-GCV/MFH

Mei Lin[a,b]*, *Junxing Huang*[a]*, *Xingmao Jiang*[c], *Jia Zhang*[b], *Hong Yu*[a], *and Jun Ye*[a], *Dongsheng Zhang*[b,d]

[a] Taizhou People's Hospital Affiliated to Nantong University, Taizhou, 225300, China,

[b] Medical School of Southeast University, Nanjing, 210009, China

[c] Key Laboratory of Advanced Catalytic Material and Technology, Changzhou University, Changzhou, 213000, China

[d] Southeast University, Jiangsu Key Laboratory For Biomaterials and Devices, Nanjing, 210009, China

Keywords: magnetic nanoparticles, radionuclide-gene therapy, magnetic fluid hyperthermia, Hepatoma, Monoclonal antibody AFP

* Both authors contributed equally to the study

Corresponding author:

Mei Lin, PhD

Taizhou People's Hospital Affiliated to Nantong University

210 Yingchun Rd.

Taizhou 225300, China

Email: l_mei@163.com

Figure S1. Schematic for the comprehensive therapy of pHRE-Egr1-HSV-TK /^{131}I-antiAFPMcAb-GCV/MFH on hepatoma

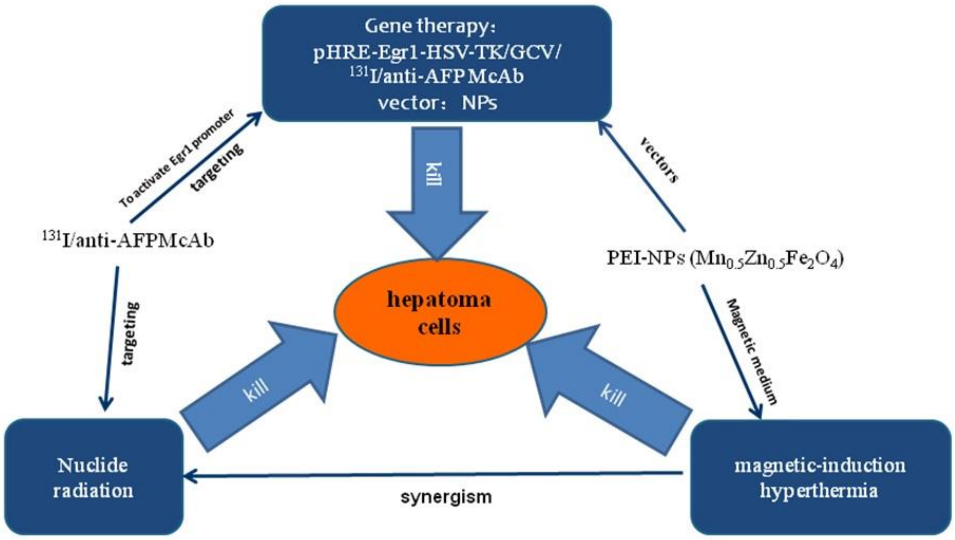

Figure S2. Heating curves of NPs *in vivo*

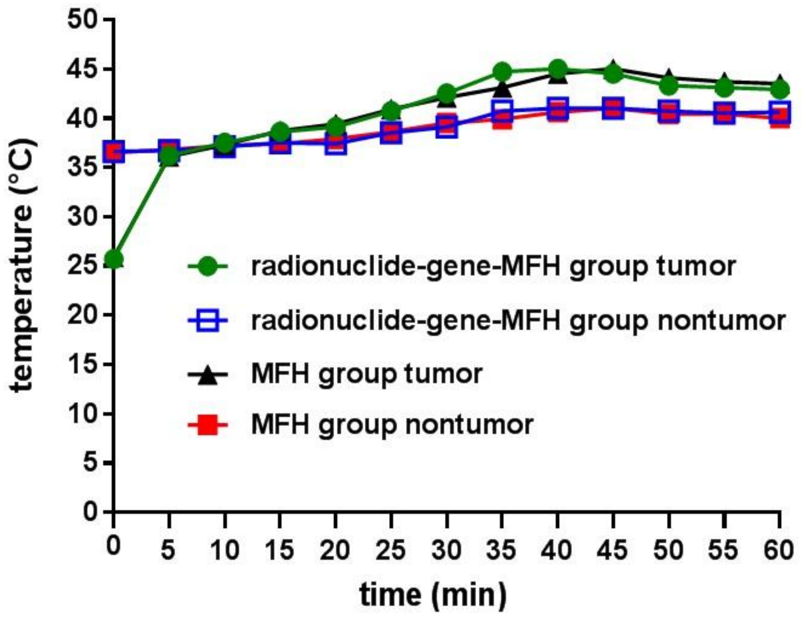

Figure S3. The tumor growth curves with different treatment

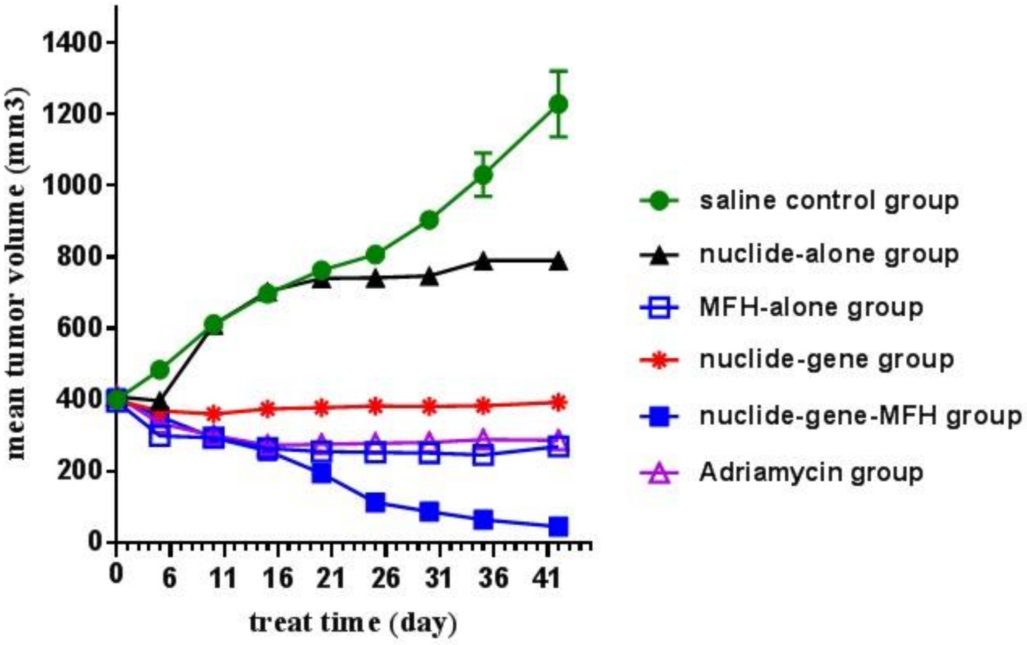

Supplement: Supplementary Information [file srep33524-s1.pdf]
